# Foot Complications in a Representative Australian Inpatient Population

**DOI:** 10.1155/2017/4138095

**Published:** 2017-10-15

**Authors:** Peter A. Lazzarini, Sheree E. Hurn, Suzanne S. Kuys, Maarten C. Kamp, Vanessa Ng, Courtney Thomas, Scott Jen, Jude Wills, Ewan M. Kinnear, Michael C. d'Emden, Lloyd F. Reed

**Affiliations:** ^1^School of Clinical Sciences, Queensland University of Technology, Brisbane, QLD, Australia; ^2^Institute of Health and Biomedical Innovation, Queensland University of Technology, Brisbane, QLD, Australia; ^3^Allied Health Research Collaborative, Metro North Hospital & Health Service, Brisbane, QLD, Australia; ^4^Wound Management Innovation Cooperative Research Centre, Brisbane, QLD, Australia; ^5^Faculty of Health Sciences, School of Physiotherapy, Australian Catholic University, Brisbane, QLD, Australia; ^6^Department of Podiatry, North West Hospital & Health Service, Mount Isa, QLD, Australia; ^7^Department of Podiatry, West Moreton Hospital & Health Service, Queensland Health, Ipswich, QLD, Australia; ^8^Department of Podiatry, Central Queensland Hospital & Health Service, Rockhampton, QLD, Australia; ^9^Department of Endocrinology & Diabetes, Metro North Hospital & Health Service, Brisbane, QLD, Australia

## Abstract

We investigated the prevalence and factors independently associated with foot complications in a representative inpatient population (adults admitted for any reason with and without diabetes). We analysed data from the *Foot disease in inpatients study*, a sample of 733 representative inpatients. Previous amputation, previous foot ulceration, peripheral arterial disease (PAD), peripheral neuropathy (PN), and foot deformity were the foot complications assessed. Sociodemographic, medical, and foot treatment history were collected. Overall, 46.0% had a foot complication with 23.9% having multiple; those with diabetes had higher prevalence of foot complications than those without diabetes (*p* < 0.01). Previous amputation (4.1%) was independently associated with previous foot ulceration, foot deformity, cerebrovascular accident, and past surgeon treatment (*p* < 0.01). Previous foot ulceration (9.8%) was associated with PN, PAD, past podiatry, and past nurse treatment (*p* < 0.02). PAD (21.0%) was associated with older age, males, indigenous people, cancer, PN, and past surgeon treatment (*p* < 0.02). PN (22.0%) was associated with older age, diabetes, mobility impairment, and PAD (*p* < 0.05). Foot deformity (22.4%) was associated with older age, mobility impairment, past podiatry treatment, and PN (*p* < 0.01). Nearly half of all inpatients had a foot complication. Those with foot complications were older, male, indigenous, had diabetes, cerebrovascular accident, mobility impairment, and other foot complications or past foot treatment.

## 1. Introduction

Active foot disease (ulcers, infection, or ischaemia) is commonly precipitated by the foot complications of previous amputations, previous foot ulcers, peripheral arterial disease (PAD), peripheral neuropathy (PN), and foot deformity in both diabetes and nondiabetes populations [1–4]. These foot complications not only increase the risk of developing active foot disease in the community but also have been found to increase the risk of developing active foot disease, falls, and pressure injuries in the inpatient setting [3, 5–8]. Thus, it seems important for clinicians, researchers, and policy makers to understand how often these foot complications present and what other factors may precipitate them in inpatient populations.

Studies investigating foot complications in inpatient populations have predominantly focused within diabetes and geriatric inpatient populations [4–9]. These studies suggest that up to 80% of diabetes inpatients, and up to 50% of geriatric inpatients, have at least one of these foot complications [4–9]. A recent systematic review of this field found previous studies have investigated either a single foot complication (such as PAD) in a representative adult inpatient population (defined as a typical hospital's inpatient population inclusive of patients admitted for any reason, with or without diabetes and of any age) or multiple foot complications in a specific inpatient population (such as all foot complications in diabetes inpatients only) [3]. This review concluded that no previous study had investigated the prevalence or factors associated with multiple foot complications in a representative inpatient population [3]. Thus, the primary aim of this study was to investigate the point prevalence and factors independently associated with foot complications in a representative adult inpatient population. A secondary aim was to investigate if there were any differences in the prevalence of foot complications between diabetes and nondiabetes inpatients.

## 2. Materials and Methods

This study was a secondary analysis of data collected from the *Foot disease in inpatients study,* a large multisite observational point prevalence study with the aim of investigating active foot disease and other foot-related conditions in a representative inpatient population [1, 2]. Previous papers from the *Foot disease in inpatients study* have investigated and reported on the outcomes of (i) primary and secondary admissions for any foot-related conditions [1] and (ii) the presence of active foot disease (ulcers, infection, and ischaemia) [2]. Thus, the design and methodology of the *Foot disease in inpatients study* that form the basis of this paper have been described in detail elsewhere [1, 2]. This paper now aims to investigate and report on the outcomes of the presence of different foot complications (previous amputations, previous foot ulcers, PAD, PN, and foot deformity).

In brief, the *Foot disease in inpatients study* investigated all adult inpatients present (in hospital at the time of the study for any medical reason) in five representative public hospitals in Queensland (considered representative of the five different categories of Australian hospitals) on one designated day and they were all invited to participate on that same day, excluding those with a cognitive deficit and those in a maternity or psychiatric ward [1]. Of a total of 1146 inpatients present on those days, 883 were eligible for the study and 733 consented to participate [1]. Those eligible participants who consented reported no age or sex differences to those who did not consent [1]. This sample of 733 inpatients has been reported to be highly reflective of the demographic, social determinant, medical history, and reason for admission characteristics reported elsewhere for representative inpatient populations present in Australia and in other developed nations [1, 2, 10]. Highly trained and tested data collectors collected all data on the 733 participants by surveying each participant to determine their self-reported medical history and physically assessing their feet to clinically diagnose any foot-related conditions [1, 11]. All data were captured on a validated data collection instrument (the Queensland Foot Disease Form) [1, 2].

The explanatory variables for this study were grouped into the domains of participant demographics (age and sex), social determinants (socioeconomic status, geographical remoteness, education levels, country of birth, and indigenous status), medical condition history (diabetes, hypertension, dyslipidaemia, myocardial infarct, cerebrovascular accident, chronic kidney disease, smoking, cancer, arthritis, depression, and acute foot trauma), self-care ability (mobility impairment, vision impairment, and main footwear worn inside and outside the home), and past foot treatment in the year prior to hospitalisation (by podiatrist, general practitioner, specialist physician, surgeon, nurse, orthotist, and other) [1, 2]. All variables have been defined in detail elsewhere [1, 2, 11].

The foot complication outcome variables for this study were previous amputation, previous foot ulceration, peripheral arterial disease (PAD), peripheral neuropathy (PN), and foot deformity [1, 2, 11–17]. All foot complication outcome variables were defined, assessed, and reported according to national and international standards for reporting research on the prevention and management of foot ulcers [13–16] and have been defined in detail elsewhere [1, 2, 11]. Previous amputation was diagnosed as a healed amputation site on the lower extremity (foot or leg) during the clinical examination [1, 2, 12–14]. Previous foot ulceration was diagnosed as a self-reported foot ulcer that had healed, and this was verified during the clinical examination [1, 2, 12–14]. PAD was diagnosed as the absence of at least one-foot pulse with a toe systolic pressure of <70 mmHg [1, 2, 11, 14–16]. Peripheral neuropathy was diagnosed as the failure to perceive the sensation of a 10-gram monofilament on at least two of three plantar forefoot sites on one foot [1, 2, 11–14, 16]. Foot deformity was diagnosed as having at least three of the following deformity characteristics on one foot: small muscle wastage, bony prominence, prominent metatarsal heads, hammer or claw toes, limited joint mobility, or Charcot deformity [1, 2, 11, 13, 17].

### 2.1. Statistical Analysis

All data were analysed using SPSS 22.0 for Windows (SPSS Inc., Chicago, IL, USA) or GraphPad Software. Descriptive statistics were used to display all variables. Prevalence with 95% confidence intervals (95% CI) was evaluated for all foot complication outcome variables. Pearson's chi-square tests and Student's *t*-tests were used to test for differences in categorical variables (proportions) and continuous variables (mean (standard deviation)), respectively. Mann–Whitney *U* tests were also used to test for differences in continuous variables (median (interquartile ranges)) if they were identified not to be normally distributed using Kolmogorov–Smirnov tests. Univariate logistic regression was used to test for crude associations between all explanatory variables and each foot complication outcome (*p* < 0.05). All explanatory variables achieving a statistical significance of *p* < 0.2, except those deemed illogical to be potentially on a causal pathway, were included in backwards stepwise multivariate logistic regression analysis for that foot complication outcome until only variables reaching statistical significance remained (*p* < 0.05) (unadjusted model) [1, 18, 19]. All omitted variables from the unadjusted models were reentered and retained in the models as confounders if the beta effect estimates of any unadjusted independent explanatory variable changed by >20% (adjusted model) [1, 18, 19]. Missing data were treated by excluding cases with any missing data in all models as the proportion of missing data cases was minimal (<5% in most cases) [1, 18, 19].

## 3. Results

Table 1 displays the prevalence proportions (95% CI) for all foot complications in all participants (*n* = 733), including diabetes participants (*n* = 172) and nondiabetes participants (*n* = 561). Overall, 336 participants (46.0% (95% CI: 42.4–49.7%)) had at least one foot complication, including 175 (23.9% (20.9–27.1%)) with multiple foot complications and 81 (11.1% (9.0–13.6%)) with a history of previous foot disease (previous amputation or foot ulceration). Diabetes participants had fewer numbers with a foot complication (*n* = 112) than nondiabetes participants (*n* = 224). Yet diabetes participants had much higher proportions of foot complications (65.5%) than nondiabetes participants (40.1%), including those with multiple foot complications (38.6% diabetes, 19.5% nondiabetes) and previous foot disease (22.1% diabetes, 7.7% nondiabetes) (all *p* < 0.001).

### 3.1. Previous Amputation

Thirty participants (4.1% (2.9–5.8%)) had a previous amputation (Table 1), including 16 (2.2% (1.3–3.6%)) with diabetes and 24 (3.3% (2.2–4.9%)) over 60 years old of total participants (Table 2). After univariate analysis, 19 explanatory variables were associated with previous amputations (all, *p* < 0.05) (Table 2). Adjusting for the identified confounder of geographical remoteness, a previous amputation was independently associated with previous foot ulcer (odds ratio (95% CI)) (22.0 (6.9–70.4)), past foot treatment by a surgeon (10.7 (2.9–40.1)), cerebrovascular accident (CVA) history (6.8 (1.9–25.2)), and foot deformity (5.6 (1.9–16.5)) (all, *p* < 0.01) (Table 3).

### 3.2. Previous Foot Ulcer

Previous foot ulcers were present in 72 participants (9.8% (7.9–12.2%)) (Table 1), including 35 (4.8% (3.4–6.6%)) with diabetes and 48 (6.6% (5.0–8.6%)) over 60 years old (Table 2). After univariate analysis, 14 explanatory variables were associated with previous foot ulcers (all, *p* < 0.05) (Table 2). Adjusting for the identified confounders of geographical remoteness and socioeconomic status, a previous foot ulcer was independently associated with past foot treatment by a nurse (18.8 (5.1–68.7)), PAD (3.9 (2.1–7.1)), PN (3.7 (2.1–6.8)), and past foot treatment by a podiatrist (2.9 (1.6–5.2)) (all, *p* < 0.01) (Table 4).

### 3.3. Peripheral Arterial Disease

Peripheral arterial disease (PAD) was present in 153 participants (21.0% (18.2–24.1%)) (Table 1), including 60 (8.2% (6.4–10.5%)) with diabetes and 121 (16.6% (14.1–19.5%)) over 60 years old (Table 5). After univariate analysis, 22 explanatory variables were associated with PAD (all, *p* < 0.05) (Table 5). Adjusting for the identified confounders of past podiatry treatment, PAD was independently associated with older age groups (41–60 years (4.7 (1.5–14.2)), 61–80 years (8.9 (3.0–26.3)), and 81+ years (12.7 (4.1–39.4))), past foot treatment by a surgeon (5.2 (2.3–11.5)), indigenous status (3.1 (1.4–7.2)), PN (2.3 (1.5–3.4)), male gender (1.7 (1.1–2.6)), and cancer history (0.5 (0.3–0.8)) (all, *p* < 0.01) (Table 6).

### 3.4. Peripheral Neuropathy

Peripheral neuropathy (PN) was present in 160 participants (22.0% (19.1–25.1%)) (Table 1), including 74 (10.2% (8.2–12.6%)) with diabetes and 124 (17.0% (14.5–19.9%)) over 60 years old (Table 5). After univariate analysis, 16 explanatory variables were associated with PN (all, *p* < 0.05) (Table 5). Adjusting for the identified confounder of geographical remoteness, PN was independently associated with older age groups (41–60 years (2.8 (1.0–7.6)), 61–80 years (4.5 (1.7–11.8)), and 81+ years (4.4 (1.6–12.3))), diabetes (3.9 (2.5–6.1)), mobility impairment (3.4 (2.2–5.2)), and PAD (2.1 (1.3–3.2)) (all, *p* < 0.05) (Table 7).

### 3.5. Foot Deformity

Foot deformity was present in 158 participants (22.4% (19.5–25.6%)) (Table 1), including 51 (7.2% (5.5–9.4%)) with diabetes and 133 (18.8% (16.2–21.9%)) over 60 years old (Table 5). After univariate analysis, 18 explanatory variables were associated with foot deformity (all, *p* < 0.05) (Table 5). No confounders were identified. Foot deformity was independently associated with older age groups (61–80 years (4.7 (1.8–12.2)) and 81+ years (5.7 (2.0–15.7))), PN (2.2 (1.4–2.4)), past foot treatment by a podiatrist (2.1 (1.4–3.1)), and mobility impairment (2.0 (1.3–3.1)) (all, *p* < 0.01) (Table 8).

## 4. Discussion

This appears to be the first study to investigate a representative inpatient population for foot complications. Our findings indicate nearly half (46%) of all inpatients had at least one foot complication that places them at risk of developing active foot disease, including nearly a quarter (24%) at higher risk with multiple foot complications and a tenth (11%) at very high risk of developing active foot disease with a history of previous foot disease. Inpatients with diabetes had significantly higher proportions of all foot complications than those without diabetes; however, interestingly, there were more patients with foot complications that did not have diabetes than did have diabetes due to the greater overall proportion of inpatients without diabetes. Foot complications in inpatients were associated with older age, males, indigenous peoples, diabetes, cerebrovascular accident (CVA) history, mobility impairment, other foot complications, and past foot treatment. Overall, these findings suggest that foot complications are relatively common in inpatient populations and also have common factors independently associated with them in both diabetes and nondiabetes inpatients.

To the best of our knowledge, the only foot complication to have been previously investigated in a representative inpatient population was PAD [3, 4]. Our 21% prevalence for PAD seemed low compared to the 29% and 36% reported in the two previous similar studies [3, 20, 21]; however, an interrogation of these studies suggests closer alignment. The previous studies used an ankle-brachial index to diagnose PAD in inpatients over 40 years, whereas our study used toe systolic pressures to diagnose PAD in inpatients over 18 years [20, 21]. Our equivalent PAD prevalence for our subgroup of inpatients over 40 years of age was 24% (149/623), and toe pressures have been found to decrease false positive PAD identification compared with the ankle brachial indices [13, 15, 16]. These methodological differences appear to explain the differences in our PAD prevalence findings compared to previous findings and suggest our findings are generalisable. We also found that diabetes patients (35%) had much higher proportions of PAD than nondiabetes patients (17%) which is also consistent with previous literature [20, 21].

Although previous amputation, previous foot ulceration, PN, and foot deformity have not been previously investigated in representative inpatient populations, our findings are generally consistent to those reported for diabetes and geriatric inpatient populations [3, 4]. Previous amputation prevalence reported by other studies were 1–8% within diabetes inpatients [4, 22, 23] and 0–7% within over 60 years old [4, 7, 24], which were similar to our findings of 9% and 6% (24/433), respectively. Previous foot ulcer prevalence reported by other studies was 12–20% within diabetes inpatients [4, 25–27] and 1–15% within over 60 years old [4, 6, 24] which again were similar to our findings of 20% and 11% (48/433), respectively. PN prevalence reported by other studies was 12–81% within diabetes inpatients [4, 9, 28] and 26% within over 60 years old [5, 6], which were again similar to our findings of 43% and 29% (124/433), respectively. Although the 43–50% foot deformity prevalence has only been previously reported in geriatric inpatients [4, 7, 29], and was much higher than our geriatric finding of 31% (133/433), this was likely explained by the different foot deformity definitions used between our study and the previous studies. Our study required at least three clinical characteristics of a foot deformity to be present to be defined as a foot deformity [13, 17], whereas previous studies required a much lower threshold of diagnosis with just one-foot deformity characteristic being required [7, 29]. The overall interpretation of our diabetes and geriatric specific inpatient findings reassures us that our foot complication prevalence findings in representative inpatient populations are plausible and generalisable.

There has also been a general lack of literature investigating independent factors associated with foot complications in representative populations (inpatient or outpatients with and without diabetes). Yet interestingly, our representative inpatient findings for factors associated with foot complications were very similar to previously reported diabetes outpatient findings, even after we adjusted for diabetes. We found previous amputation was most strongly associated with previous foot ulcers which have been consistently identified in the diabetes literature to be the major precipitating risk factor for amputation [12–14, 16, 17, 30]. Other factors identified in our study were foot deformity, CVA history, and past foot treatment by a surgeon. The association with foot deformity is most likely explained by a minor amputation procedure often producing a foot deformity in the remaining partial foot and a major amputation often precipitating a compensatory foot deformity in the remaining contralateral foot [13, 17]. A CVA history has also been identified in other recent studies [30, 31] and may be explained by the similar macrovascular pathophysiology that occurs in both CVA and PAD which can subsequently result in amputation [30, 31]. Lastly, past foot treatment by a surgeon in the previous year is perhaps not surprising considering amputation procedures can only be performed by surgeons; however, we only captured past foot treatment for the year prior to hospitalisation and did not record the duration since the previous amputation was performed. Thus, further research would be required to determine how long people with a previous amputation maintain ongoing foot treatment with their surgeon after their procedure.

The independent factors we identified to be associated with previous foot ulceration were also consistent with those reported in the diabetes outpatient literature [12–14, 17, 32]. Our findings indicate that PAD, PN, and past foot treatment factors are important factors associated with foot ulceration, regardless of diabetes status [13, 17, 32]. Furthermore, a recent study also identified that PAD and PN were independently associated with active foot ulcers, regardless of diabetes; however, that study identified an association with past foot treatment by a surgeon rather than a podiatrist or nurse [2]. This adds weight to previous recommendations that best practice guidelines must emphasise the need for a podiatrist and nurse, in conjunction with a surgeon, to be part of the recommended outpatient multidisciplinary foot team to prevent foot ulcer inpatient admissions in diabetes and nondiabetes patients, rather than wait until the foot ulcer has healed to seek podiatry and nursing foot treatment as our findings suggest happens [13, 33, 34]. Nevertheless, these findings suggest, in both diabetes and nondiabetes populations, that these foot complications significantly increase the risk of developing future active foot disease (ulcers, infection, or ischaemia) which in turn increases the risk of potential hospitalisation and amputation.

PAD in our study was independently associated with older age, male gender, and PN, which is consistent with previous outpatient literature [20, 21, 35]. Furthermore, our study identified Australian indigenous peoples as an independent factor associated with PAD which has also been identified by two previous diabetes-related Australian studies [36, 37]. The independent associated factor of past surgical treatment for PAD is a welcome finding and suggests patients with PAD are being regularly assessed by vascular surgeons as recommended by best practice guidelines [13, 15, 16], whereas our finding that a cancer history decreased the likelihood of having PAD is potentially a novel finding. It is perhaps most likely explained by cancer sufferers being hospitalised at younger ages [10], but more likely because our definition of cancer history was broad and included any cancers in the participant's history. Thus, it is recommended that any further research into this association captures data on different cancer types, severity, durations, and treatments to determine if the association is with particular types of cancers or treatments.

PN was found to be independently associated with older age, diabetes, and mobility impairment in our study, again all of which have been reported in the diabetes outpatient literature [32, 38]. In contrast to best practice guideline recommendations that people with PN should receive regular foot monitoring by a podiatrist to prevent active foot disease, falls, or pressure injuries [13, 33], our study found no past foot treatment variables were independently associated with PN. PN has been reported to be the most important foot complication that precipitates the development of active foot disease, falls, and pressure injuries [3, 5–8]. Thus, our findings that one in every five inpatients, including nearly one in every two diabetes inpatients, has PN and is unlikely to have received any past foot treatment are concerning and highlight that further strategies are necessary to identify and monitor these patients in both the inpatient and outpatient settings [13, 33, 34]. The independent factors for foot deformity of older age, PN, mobility impairment, and past podiatry treatment identified in our inpatient study are also consistent with similar outpatient literature [17, 38–40]. The association between foot deformity and PN has been consistently identified in the diabetes literature, and our findings suggest this link may be of similar importance in nondiabetes patients and should be investigated further in the future [17, 38–40].

Our overall findings suggest that foot complications that have been commonly reported to precipitate the development of active foot disease in the community are also present frequently in the inpatient population and have common factors independently associated with them, regardless of diabetes. Further research is recommended to more precisely determine the causal relationships for foot complications in nondiabetes populations in particular. It is recommended that policy makers and clinicians adopt simple hospital triage procedures that identify inpatients with these foot complications early to ensure that they do not develop into future active foot disease, falls, or pressure injuries whilst in hospital [1, 2, 33, 34]. These procedures could be as simple as questioning all inpatients on admission, particularly those with diabetes or over 60 years of age, as to their previous foot disease history or using simple screening tools to identify PAD, PN, and foot deformity [1, 2, 13, 34]. Nevertheless, clinicians and policy makers should continue to recommend inpatients identified with foot complications be assessed and managed whilst in an hospital and discharged to an outpatient multidisciplinary foot team for ongoing management in order to prevent potential hospitalisation from active foot disease, falls, and pressure injuries in the future [1, 2, 33, 34].

Additionally, although the independent associations between past foot treatment and most foot complications appear encouraging in our findings, this was not the case for PN. It could be argued that PN is the most critical foot complication that leads to active foot disease, falls, and pressure injuries, and thus, best practice guidelines need to better highlight that patients with PN require ongoing monitoring to ensure they can identify problems early to prevent possible future hospitalisation [13, 17, 32–34]. Lastly, it is recommended that policy makers incorporate these foot complications into their existing inpatient bedside audit programs alongside general diabetes, falls, and pressure injury bedside audit programs [2, 10, 26]. This should enable efficient monitoring of the influence of these foot complications on inpatient populations in the future and particularly their impact on inpatient adverse events [1, 2].

### 4.1. Strengths and Limitations

This study has a number of strengths and limitations which have been discussed elsewhere [1, 2]. In brief, the strengths were this study investigated a highly representative Australian inpatient population [1, 2, 10]; data collectors were highly experienced, trained, and reliable in collecting validated and internationally agreed definitions of clinically diagnosed foot complications [11, 14]; and multivariate logistic regression models were used to adjust for confounding variables [1, 18, 19]. The limitations were this study was a cross-sectional study and was unable to test for causal relationships; excluded a large number of older cognitively impaired patients which may have led to a more conservative prevalence estimate of foot complications; had to aggregate minor and major previous amputations which are arguably the result of different causal pathways due to small numbers of both; did not exclude patients with active foot disease; and was a secondary analysis of a large dataset [1, 2] which increases the likelihood of type 1 statistical error [18, 19].

## 5. Conclusions

This study was the first to investigate multiple foot complications in a representative inpatient population. It identified that half of all inpatients had at least one-foot complication, with a quarter having multiple foot complications, which have been reported to be risk factors for the development active foot disease, pressure injuries, or falls whilst in hospital. The findings of this study suggest that regardless of having diabetes or not, common factors precipitate these foot complications. It is recommended that all inpatients are screened for these common foot complications on admission, particularly those with diabetes, and are managed accordingly to potentially prevent the large burden that foot disease already imposes on inpatient and outpatient populations.

## Figures and Tables

**Table 1:** Proportion of the diabetes and nondiabetes participants with foot complications.

Foot complication	All *n* (% (95% CI))	Diabetes *n* (% (95% CI))	Nondiabetes *n* (% (95% CI))	*p* value
Participants	733	172	561	
Foot complication(s)^a^	336 (46.0% (42.4–49.7))	112 (65.5% (58.1–72.2)	224 (40.1% (36.2–44.3))	<0.001^∗^
Multiple foot complications^b^	175 (23.9% (20.9–27.1))	66 (38.6% (31.6–46.1))	109 (19.5% (16.4–23.0))	<0.001^∗^
Previous foot disease^c^	81 (11.1% (9.0–13.6))	38 (22.1% (16.5–28.9))	43 (7.7% (5.7–10.2))	<0.001^∗^
Previous amputation	30 (4.1% (2.9–5.8))	16 (9.3% (5.7–14.7))	14 (2.5% (1.4–4.2)))	<0.001^∗^
Previous foot ulcer	72 (9.8% (7.9–12.2)	35 (20.3% (15.0–27.0))	37 (6.6% (4.8–9.0)))	<0.001^∗^
Peripheral arterial disease	153 (21.0% (18.2–24.1))	60 (35.1% (28.3–42.5))	93 (16.7% (13.8–20.0))	<0.001^∗^
Peripheral neuropathy	160 (22.0% (19.1–25.1))	74 (43.3% (36.1–50.8))	86 (15.4% (12.6–18.6))	<0.001^∗^
Foot deformity	158 (22.4% (19.5–25.6))	51 (30.5% (24.0–37.9))	107 (19.9% (16.7–23.5))	0.004^∗^

^∗^ *p* < 0.05. ^a^Participants with at least one foot complication. ^b^Participants with two or more foot complications. ^c^Participants with previous foot disease (either previous foot ulcer or previous amputation); CI: confidence interval.

**Table 2:** Participant characteristics and univariate analysis for previous amputation and previous foot ulcer.

Variables	All	Previous amputation			Previous foot ulcer		
		*n* (%)	Odds ratio (95% CI)	*p* value	*n* (%)	Odds ratio (95% CI)	*p* value
Participants	733	30 / 731 (4.1%)			72/731 (9.8%)		
*Demographics*							
Age: mean (SD) years	62.0 (18.6)	71.4 (11.1)	1.04 (1.01–1.06)	0.006^∗∗^	65.8 (15.6)	1.01 (1.00–1.03)	0.074^∗^
Age: median (IQR) years	65 (50–76)	72 (66–79)		0.006^∗∗^	69 (57–76)		0.128^∗^
Age groups				NA			0.110
18–40 years	110 (15.0%)	0	1.00		5 (6.9%)	1.00	
41–60 years	188 (25.7%)	6 (20.0%)			19 (26.4%)	2.38 (0.86–6.55)	0.095
61–80 years	316 (43.2%)	17 (56.7%)			39 (54.2%)	2.97 (1.14–7.73)	0.026
81+ years	117 (16.0%)	7 (23.3%)			9 (12.5%)	1.75 (0.57–5.39)	0.330
Male sex	408 (55.8)	19 (63.3%)	1.38 (0.65–2.95)	0.400	46 (63.9%)	1.46 (0.88–2.42)	0.142^∗^

*Social determinants*							
Socioeconomic status	711			0.638			0.064^∗^
Most disadvantaged	102 (14.4%)	6 (20.7%)	1.00		16 (22.9%)	1.00	
Second most disadvantaged	159 (22.4%)	7 (24.1%)	0.74 (0.24–2.26)	0.593	17 (24.3%)	0.64 (0.31–1.34)	0.239
Middle	98 (13.8%)	2 (6.9%)	0.33 (0.07–1.69)	0.185	4 (5.7%)	0.23 (0.07–0.72)	0.011
Second least disadvantaged	240 (33.8%)	11 (37.9%)	0.78 (0.28–2.16)	0.626	26 (37.1%)	0.66 (0.34–1.28)	0.218
Least disadvantaged	112 (15.8%)	3 (10.3%)	0.44 (0.11–1.81)	0.255	7 (10.0%)	0.36 (0.14–0.91)	0.031
Geographic remoteness	711			0.589			0.304
Major city	435 (61.2%)	18 (62.1%)	1.00		41 (58.6%)	1.00	
Inner regional area	153 (21.5%)	4 (13.8%)	0.62 (0.21–1.86)	0.392	15 (21.4%)	1.04 (0.56–1.94)	0.904
Outer regional area	66 (9.3%)	5 (17.2%)	1.89 (0.68–5.28)	0.224	12 (17.1%)	2.13 (1.05–4.29)	0.036
Remote area	30 (4.2%)	1 (3.4%)	0.80 (0.10–6.17)	0.826	0	0	NA
Very remote area	27 (3.8%)	1 (3.4%)	0.89 (0.11–6.90)	0.909	2 (2.9%)	0.77 (0.18–3.35)	0.722
<10-year education level	395 (54.0%)	19 (63.3%)	1.48 (0.70–3.16)	0.307	41 (56.9%)	1.13 (0.69–1.85)	0.621
Indigenous	34 (4.6%)	1 (3.3%)	0.70 (0.09–5.27)	0.727	4 (5.6%)	1.23 (0.42–3.60)	0.704
Born overseas	161 (22.0%)	5 (16.7%)	0.70 (0.26–1.85)	0.467	11 (15.3%)	0.61 (0.31–1.19)	0.146^∗^

*Medical condition history*							
Diabetes	172 (23.5%)	16 (53.3%)	3.99 (1.91–8.36)	<0.001^∗∗^	35 (48.6%)	3.60 (2.19–5.94)	<0.001^∗∗^
Hypertension	359 (49.0%)	21 (70.0%)	2.54 (1.15–5.61)	0.022^∗∗^	38 (52.8%)	1.18 (0.73–1.93)	0.497
Dyslipidaemia	234 (31.9%)	12 (40.0%)	1.44 (0.68–3.04)	0.341	27 (37.5%)	1.31 (0.79–2.17)	0.294
Myocardial infarct	146 (19.9%)	12 (40.0%)	2.82 (1.33–6.00)	0.007^∗∗^	17 (23.6%)	1.27 (0.71–2.26)	0.417
Cerebrovascular accident	85 (11.6%)	9 (30.0%)	3.52 (1.59–7.97)	0.002^∗∗^	8 (11.1%)	0.95 (0.44–2.05)	0.885
Chronic kidney disease	89 (12.1%)	11 (36.7%)	4.62 (2.12–10.08)	<0.001^∗∗^	19 (26.4%)	3.02 (1.69–5.39)	<0.001^∗∗^
Smoker	104 (14.2%)	3 (10.0%)	0.66 (0.20–2.22)	0.501	12 (16.7%)	1.23 (0.64–2.38)	0.533
Ex-smoker	304 (41.5%)	14 (46.7%)	1.25 (0.60–2.60)	0.554	28 (38.9%)	0.89 (0.54–1.46)	0.642
Cancer	174 (23.7%)	8 (26.7%)	1.17 (0.51–2.68)	0.707	17 (23.6%)	0.99 (0.56–1.75)	0.968
Arthritis	274 (37.4%)	18 (60.0%)	2.64 (1.25–5.57)	0.011^∗∗^	41 (56.9%)	2.43 (1.49–3.99)	<0.001^∗∗^
Depression	191 (26.1%)	6 (20.0%)	0.70 (0.28–1.75)	0.447	21 (29.2%)	1.18 (0.69–2.03)	0.537
Acute foot trauma	26 (3.5%)	1 (3.3%)	0.93 (0.12–7.12)	0.946	7 (9.7%)	3.63 (1.47–8.95)	0.005^∗∗^

*Self-care ability*							
Mobility impairment	242 (33.2%)	21 (70.0%)	5.07 (2.29–11.25)	<0.001^∗∗^	38 (52.8%)	2.48 (1.52–4.05)	<0.001^∗∗^
Vision impairment	110 (15.1%)	12 (40.0%)	4.09 (1.91–8.75)	<0.001^∗∗^	20 (27.8%)	2.42 (1.38–4.25)	0.002^∗∗^
Footwear worn: inside				0.158^∗^			0.580
Low-risk footwear	81 (11.1%)	6 (20.7%)	1.00		11 (15.5%)	1.00	
Moderate-risk footwear	263 (36.1%)	13 (44.8%)	0.65 (0.24–1.77)	0.399	27 (38.0%)	0.73 (0.34–1.54)	0.407
High-risk footwear	139 (19.1%)	2 (6.9%)	0.18 (0.04–0.93)	0.041	12 (16.9%)	0.61 (0.25–1.45)	0.259
No footwear worn	245 (33.7%)	8 (27.6%)	0.42 (0.14–1.26)	0.121	21 (29.6%)	0.60 (0.27–1.30)	0.193
Footwear worn: outside				0.116^∗^			0.235
Low-risk footwear	386 (53.2%)	21 (75.0%)	1.00		36 (50.7%)	1.00	
Moderate-risk footwear	75 (10.3%)	1 (3.6%)	0.23 (0.03–1.77)	0.159	11 (15.5%)	1.67 (0.81–3.44)	0.168
High-risk footwear	250 (34.4%)	5 (17.9%)	0.35 (0.13–0.95)	0.039	21 (29.6%)	0.89 (0.51–1.56)	0.682
No footwear worn	15 (2.1%)	1 (3.6%)	1.24 (0.16–9.87)	0.840	3 (4.2%)	2.42 (0.65–8.99)	0.186

*Past foot treatment*							
Yes	256 (34.9%)	22 (73.3%)	5.52 (2.42–12.60)	<0.001^∗∗^	56 (77.8%)	8.03 (4.50–14.35)	<0.001^∗∗^
Podiatry	180 (24.6%)	18 (60.0%)	50.3 (2.37–10.67)	<0.001^∗∗^	41 (56.9%)	4.95 (2.99–8.18)	<0.001^∗∗^
GP	93 (12.7%)	14 (46.7%)	6.89 (3.24–14.65)	<0.001^∗∗^	27 (37.5%)	5.39 (3.14–9.26)	<0.001^∗∗^
Surgeon	36 (4.9%)	14 (46.7%)	27.01 (11.73–62.15)	<0.001^∗∗^	17 (23.6%)	10.41 (5.12–21.18)	<0.001^∗∗^
Physician	21 (2.9%)	5 (16.7%)	8.56 (2.91–25.23)	<0.001^∗∗^	7 (9.7%)	4.96 (1.93–12.73)	0.001^∗∗^
Nurse	20 (2.7%)	7 (23.3%)	16.11 (5.88–44.15)	<0.001^∗∗^	12 (16.7%)	16.28 (6.40–41.37)	<0.001^∗∗^
Orthotist	4 (0.5%)	3 (10.0%)	77.78 (7.83–772.35)	<0.001^∗∗^	2 (2.8%)	9.39 (1.30–67.67)	0.026^∗∗^
Other	9 (1.2%)	0	0	NA	1 (1.4%)	1.15 (0.14–9.30)	0.898

*Foot disease history*							
Previous foot ulcer	72 (9.8%)	21 (70.0%)	30.33 (13.20–69.72)	<0.001^∗∗^	—	—	—

*Foot risk factors*							
Peripheral neuropathy	160 (22.0%)	21 (72.4%)	10.65 (4.62–24.56)	<0.001^∗∗^	39 (54.9%)	5.39 (3.24–8.95)	<0.001^∗∗^
PAD	153 (21.0%)	20 (69.0%)	9.44 (4.20–21.20)	<0.001^∗∗^	40 (56.3%)	6.20 (3.72–10.34)	<0.001^∗∗^
Foot deformity	158 (22.4%)	17 (65.4%)	7.27 (3.17–16.66)	<0.001^∗∗^	29 (42.0%)	2.85 (1.70–4.77)	<0.001^∗∗^

^∗^ *p* < 0.2; ^∗∗^*p* < 0.05; CI: confidence interval; GP: general practitioner; IQR: interquartile range; PAD: peripheral arterial disease; SD: standard deviation.

**Table 3:** Independent factors associated with previous amputations (odds ratios [95% CI]).

Risk factor	Unadjusted	*p* value	Adjusted^a^	*p* value
CVA history	4.83 (1.48–15.74)	0.009^∗^	6.85 [1.86–25.21]	0.004^∗^
Previous foot ulcer	17.92 (6.51–49.29)	<0.001^∗^	22.01 [6.89–70.38]	<0.001^∗^
Foot deformity	4.50 (1.70–11.90)	0.002^∗^	5.59 [1.89–16.55]	0.002^∗^
Surgeon past foot treatment	8.09 (2.50–26.20)	<0.001^∗^	10.73 [2.87–40.15]	<0.001^∗^

Model 1 results	Pseudo *R*^2^: 0.447 Omnibus: df = 4, *p* < 0.001	Missing: 29 (4.0%); H&L: *p* = 0.955	Pseudo *R*^2^: 0.516 Omnibus: df = 8, *p* < 0.001	Missing: 50 (6.8%); H&L: *p* = 0.628

^∗^ *p* < 0.05. ^a^Adjusted for identified confounder of geographical remoteness; pseudo *R*^2^: Nagelkerke *R*^2^; omnibus: omnibus tests of model coefficients; df: degrees of freedom; missing: excluded cases with any missing data; H&L: Hosmer and Lemeshow test; CVA: cardiovascular accident.

**Table 4:** Independent factors associated with previous foot ulcers (odds ratios [95% CI]).

Risk factor	Unadjusted	*p* value	Adjusted^a^	*p* value
Vision impairment	2.10 (1.09–4.03)	0.026^∗^	1.89 (0.95–3.77)	0.069
PN	3.17 (1.81–5.56)	<0.001^∗^	3.75 (2.06–6.85)	<0.001^∗^
PAD	3.77 (2.15–6.62)	<0.001^∗^	3.88 [2.14–7.06]	<0.001^∗^
Podiatry past foot treatment	3.16 (1.80–5.55)	<0.001^∗^	2.88 (1.59–5.22)	<0.001^∗^
Nurse past foot treatment	8.45 (2.88–24.84)	<0.001^∗^	18.80 (5.15–68.66)	<0.001^∗^

Model 1 results	Pseudo *R*^2^: 0.305 Omnibus: df = 5, *p* < 0.001	Missing: 9 (1.2%); H&L: *p* = 0.154	Pseudo *R*^2^: 0.364 Omnibus: df = 13, *p* < 0.001	Missing: 31 (4.2%); H&L: *p* = 0.601

^∗^ *p* < 0.05. ^a^Adjusted for identified confounders of geographical remoteness and socioeconomic status; pseudo *R*^2^: Nagelkerke *R*^2^; omnibus: omnibus tests of model coefficients; df: degrees of freedom; missing: excluded cases with any missing data; H&L: Hosmer and Lemeshow test; PAD: peripheral arterial disease; PN: peripheral neuropathy.

**Table 5:** Participant characteristics and univariate analysis for peripheral arterial disease, peripheral neuropathy, and foot deformity.

Variables	All	Peripheral arterial disease			Peripheral neuropathy			Foot deformity		
		*n* (%)	Odds ratio (95% CI)	*p* value	*n* (%)	Odds ratio (95% CI)	*p* value	*n* (%)	Odds ratio (95% CI)	*p* value
Participants	733	153/728 (21.0%)			160/728 (22.0%)			158/706 (22.4%)		
*Demographics*										
Age: mean (SD) years	62.0 (18.6)	70.5 (13.3)	1.04 (1.03–1.05)	<0.001^∗∗^	70.1 (14.1)	1.04 (1.02–1.05)	<0.001^∗∗^	72.3 (14.4)	1.05 (1.04–1.06)	<0.001^∗∗^
Age: median (IQR) years	65 (50–76)	73 (63–81)		<0.001^∗∗^	73 (62–80)		<0.001^∗∗^	75 (66–82)		<0.001^∗∗^
Age groups				<0.001^∗^			<0.001^∗∗^			<0.001^∗∗^
18–40 years	110 (15.0%)	4 (2.6%)	1.00		6 (3.8%)	1.00		5 (3.2%)	1.00	
41–60 years	188 (25.7%)	28 (18.3%)	4.70 (1.60–13.78)	0.005	29 (18.2%)	3.22 (1.29–8.03)	0.012	19 (12.1%)	2.33 (0.84–6.43)	0.103
61–80 years	316 (43.2%)	82 (53.6%)	9.41 (3.36–26.34)	<0.001	87 (54.7%)	6.64 (2.81–15.69)	<0.001	88 (56.1%)	7.84 (3.09–19.91)	<0.001^∗∗^
81+ years	117 (16.0%)	39 (25.5%)	13.25 (4.55–38.62)	<0.001	37 (23.3%)	8.02 (3.23–19.93)	<0.001	45 (28.7%)	12.60 (4.76–33.36)	<0.001^∗∗^
Male sex	408 (55.8)	97 (63.4%)	1.48 (1.03–2.14)	0.037^∗∗^	93 (58.1%)	1.12 (0.79–1.60)	0.525	74 (46.8%)	0.62 (0.44–0.89)	0.009^∗∗^

*Social determinants*										
Socioeconomic status	711			0.020^∗∗^			0.798			0.274
Most disadvantaged	102 (14.4%)	32 (21.8%)	1.00		24 (15.5%)	1.00		22 (14.5%)	1.00	
Second most disadvantaged	159 (22.4%)	34 (23.1%)	0.59 (0.33–1.04)	0.066	34 (21.9%)	0.88 (0.48–1.59)	0.661	35 (23.0%)	1.03 (0.56–1.89)	0.927
Middle	98 (13.8%)	13 (8.8%)	0.33 (0.16–0.67)	0.002	21 (13.5%)	0.86 (0.44–1.68)	0.666	16 (10.5%)	0.67 (0.33–1.36)	0.264
Second least disadvantaged	240 (33.8%)	49 (33.3%)	0.55 (0.32–0.93)	0.025	56 (36.1%)	0.97 (9.56–1.68)	0.910	49 (32.2%)	0.89 (0.51–1.58)	0.698
Least disadvantaged	112 (15.8%)	19 (12.9%)	0.43 (0.23–0.83)	0.012	20 (12.9%)	0.69 (0.35–1.34)	0.272	30 (19.7%)	1.40 (0.74–2.65)	0.300
Geographic remoteness	711			0.604			0.556			0.180^∗^
Major city	435 (61.2%)	87 (59.2%)	1.00		98 (63.2%)	1.00		103 (67.8%)	1.00	
Inner regional area	153 (21.5%)	30 (20.4%)	0.98 (0.62–1.56)	0.943	32 (20.6%)	0.93 (0.59–1.46)	0.742	28 (18.4%)	0.73 (0.46–1.17)	0.196
Outer regional area	66 (9.3%)	17 (11.6%)	1.38 (0.76–2.51)	0.297	10 (15.2%)	0.61 (0.30–1.24)	0.173	15 (9.9%)	0.92 (0.50–1.71)	0.793
Remote area	30 (4.2%)	5 (3.4%)	0.79 (0.30–2.13)	0.646	9 (5.8%)	1.47 (0.65–3.30)	0.357	2 (1.3%)	0.23 (0.05–0.97)	0.046
Very remote area	27 (3.8%)	8 (5.4%)	1.67 (0.71–3.94)	0.242	6 (3.9%)	0.98 (0.38–2.49)	0.961	4 (2.6%)	0.53 (0.18–1.58)	0.256
<10-year education level	395 (54.0%)	97 (63.4%)	1.64 (1.14–2.37)	0.008^∗∗^	92 (57.9%)	1.23 (0.86–1.76)	0.252	98 (62.0%)	1.54 (1.07–2.21)	0.020^∗∗^
Indigenous	34 (4.6%)	12 (7.9%)	2.16 (1.04–4.46)	0.039^∗∗^	9 (5.6%)	1.29 (0.59–2.83)	0.521	7 (4.5%)	0.94 (0.40–2.20)	0.881
Born overseas	161 (22.0%)	33 (21.6%)	0.96 (0.62–1.47)	0.839	29 (18.1%)	0.73 (0.47–1.14)	0.164^∗^	35 (22.3%)	1.02 (0.67–1.56)	0.925

*Medical condition history*										
Diabetes	172 (23.5%)	60 (39.2%)	2.70 (1.84–3.96)	<0.001^∗∗^	74 (46.3%)	4.18 (2.86–6.11)	<0.001^∗∗^	51 (32.3%)	1.76 (1.20–2.63)	0.004^∗∗^
Hypertension	359 (49.0%)	97 (63.4%)	2.10 (1.45–3.03)	<0.001^∗∗^	92 (57.5%)	1.55 (1.09–2.20)	0.016^∗∗^	95 (60.1%)	1.75 (1.22–2.50)	0.002^∗∗^
Dyslipidaemia	234 (31.9%)	66 (43.1%)	1.87 (1.30–2.70)	0.001^∗∗^	65 (40.6%)	1.63 (1.13–2.34)	0.008^∗∗^	53 (33.5%)	1.09 (0.75–1.58)	0.671
Myocardial infarct	146 (19.9%)	42 (27.5%)	1.73 (1.15–2.62)	0.009^∗∗^	38 (23.8%)	1.33 (0.87–2.02)	0.187^∗^	43 (27.2%)	1.66 (1.10–2.50)	0.016^∗∗^
Cerebrovascular accident	85 (11.6%)	29 (19.0%)	2.17 (1.33–3.54)	0.002^∗∗^	24 (15.0%)	1.47 (0.88–2.44)	0.140^∗^	21 (13.3%)	1.22 (0.72–2.08)	0.456
Chronic kidney disease	89 (12.1%)	38 (24.8%)	3.47 (2.17–5.54)	<0.001^∗∗^	32 (20.0%)	2.24 (1.40–3.61)	0.001^∗∗^	28 (17.7%)	1.79 (1.09–2.91)	0.020^∗∗^
Smoker	104 (14.2%)	21 (13.7%)	0.96 (0.57–1.60)	0.866	18 (11.3%)	0.72 (0.42–1.24)	0.235	10 (6.3%)	0.35 (0.18–0.69)	0.002^∗∗^
Ex-smoker	304 (41.5%)	69 (45.1%)	1.21 (0.84–1.73)	0.308	66 (41.3%)	0.98 (0.69–1.40)	0.914	68 (43.0%)	1.07 (0.75–1.53)	0.717
Cancer	174 (23.7%)	29 (19.0%)	0.71 (0.45–1.10)	0.127^∗^	45 (28.1%)	1.35 (0.90–2.00)	0.143^∗^	39 (24.7%)	1.09 (0.72–1.64)	0.694
Arthritis	274 (37.4%)	77 (50.3%)	1.99 (1.39–2.86)	<0.001^∗∗^	73 (45.6%)	1.57 (1.10–2.24)	0.013^∗∗^	82 (51.9%)	2.24 (1.55–3.19)	<0.001^∗∗^
Depression	191 (26.1%)	36 (23.5%)	0.85 (0.56–1.29)	0.440	41 (25.6%)	0.97 (0.65–1.45)	0.877	44 (27.8%)	1.09 (0.74–1.63)	0.660
Acute foot trauma	26 (3.5%)	5 (3.3%)	0.89 (0.33–2.40)	0.820	9 (5.6%)	1.93 (0.84–4.42)	0.119^∗^	6 (3.8%)	1.04 (0.41–2.64)	0.931

*Self-care ability*										
Mobility impairment	242 (33.2%)	78 (51.0%)	2.66 (1.84–3.83)	<0.001^∗∗^	95 (59.4%)	4.23 (2.93–6.12)	<0.001^∗∗^	89 (56.3%)	3.59 (2.49–5.19)	<0.001^∗∗^
Vision impairment	110 (15.1%)	37 (24.2%)	2.22 (1.42–3.46)	<0.001^∗∗^	33 (20.6%)	1.68 (1.07–2.64)	0.026^∗∗^	37 (23.6%)	2.14 (1.37–3.34)	0.001^∗∗^
Footwear worn: inside				0.002^∗∗^			0.006^∗∗^			0.007^∗∗^
Low-risk footwear	81 (11.1%)	19 (12.4%)	1.00		27 (17.0%)	1.00		22 (13.9%)	1.00	
Moderate-risk footwear	263 (36.1%)	74 (48.4%)	1.28 (0.72–2.28)	0.408	66 (41.5%)	0.67 (0.39–1.16)	0.151	73 (46.2%)	1.07 (0.61–1.87)	0.813
High-risk footwear	139 (19.1%)	19 (12.4%)	0.53 (0.26–1.06)	0.074	25 (15.7%)	0.44 (0.24–0.83)	0.012	23 (14.6%)	0.56 (0.29–1.08)	0.083
No footwear worn	245 (33.7%)	41 (26.8%)	0.66 (0.36–1.21)	0.178	41 (25.8%)	0.40 (0.23–0.71)	0.002	40 (25.3%)	0.56 (0.31–1.01)	0.054
Footwear worn: outside				0.065^∗^			0.015^∗∗^			0.316
Low-risk footwear	386 (53.2%)	89 (58.6%)	1.00		91 (57.2%)	1.00		85 (54.1%)	1.00	
Moderate-risk footwear	75 (10.3%)	19 (12.5%)	1.13 (0.64–2.01)	0.670	16 (10.1%)	0.88 (0.48–1.60)	0.674	22 (14.0%)	1.43 (0.82–2.49)	0.207
High-risk footwear	250 (34.4%)	39 (25.7%)	0.62 (0.41–0.94)	0.026	44 (27.7%)	0.70 (0.47–1.05)	0.081	47 (29.9%)	0.81 (0.55–1.21)	0.313
No footwear worn	15 (2.1%)	5 (3.3%)	1.67 (0.56–5.01)	0.361	8 (5.0%)	3.71 (1.31–10.50)	0.014	3 (1.9%)	0.92 (0.25–3.38)	0.901

*Past foot treatment*										
Yes	256 (34.9%)	83 (54.2%)	2.80 (1.95–4.04)	<0.001^∗∗^	86 (53.8%)	2.77 (1.93–3.96)	<0.001^∗∗^	86 (54.4%)	2.80 (1.95–4.02)	<0.001^∗∗^
Podiatry	180 (24.6%)	62 (40.5%)	2.67 (1.82–3.91)	<0.001^∗∗^	67 (41.9%)	2.93 (2.01–4.27)	<0.001^∗∗^	73 (46.2%)	3.71 (2.54–5.42)	<0.001^∗∗^
GP	93 (12.7%)	36 (23.5%)	2.85 (1.79–4.54)	<0.001^∗∗^	37 (23.1%)	2.81 (1.77–4.45)	<0.001^∗∗^	25 (15.8%)	1.42 (0.86–2.25)	0.168^∗^
Surgeon	36 (4.9%)	20 (13.1%)	5.61 (2.80–11.26)	<0.001^∗∗^	18 (11.3%)	4.11 (2.07–8.18)	<0.001^∗∗^	14 (8.9%)	2.71 (1.33–5.53)	0.006^∗∗^
Physician	21 (2.9%)	9 (5.9%)	2.93 (1.21–7.09)	0.017^∗∗^	8 (5.0%)	2.25 (0.92–5.52)	0.078^∗^	9 (5.7%)	2.95 (1.20–7.25)	0.018^∗∗^
Nurse	20 (2.7%)	10 (6.5%)	3.95 (1.61–9.68)	0.003^∗∗^	11 (6.9%)	4.59 (1.87–11.27)	0.001^∗∗^	9 (5.7%)	3.25 (1.30–8.14)	0.012^∗∗^
Orthotist	4 (0.5%)	1 (0.7%)	1.25 (0.13–12.14)	0.845	3 (1.9%)	10.83 (1.12–104.88)	0.040	1 (0.6%)	1.74 (0.16–19.30)	0.652
Other	9 (1.2%)	2 (1.3%)	1.08 (0.22–5.23)	0.929	1 (0.6%)	0.44 (0.06–3.55)	0.441	3 (1.9%)	1.75 (0.43–7.07)	0.433

*Foot risk factors*										
Peripheral neuropathy	160 (22.0%)	62 (40.5%)	3.35 (2.27–4.95)	<0.001^∗∗^	—	—	—	66 (42.0%)	3.69 (2.50–5.45)	<0.001^∗∗^
PAD	153 (21.0%)	—	—	—	62 (39.0%)	3.35 (2.27–4.95)	<0.001^∗∗^	53 (33.8%)	2.43 (1.63–3.62)	<0.001^∗∗^
Foot deformity	158 (22.4%)	52 (35.8%)	2.43 (1.63–3.62)	<0.001^∗∗^^a^	66 (42.3%)	3.69 (2.50–5.45)	<0.001^∗∗^^a^	—	—	—

^∗^ *p* < 0.2; ^∗∗^*p* < 0.05. ^a^Explanatory variable excluded from multivariate model as considered not on causal pathway for outcome; CI: confidence interval; GP: general practitioner; IQR: interquartile range; PAD: peripheral arterial disease; SD: standard deviation.

**Table 6:** Independent factors associated with peripheral arterial disease (odds ratios [95% CI]).

Risk factor	Unadjusted	*p* value	Adjusted^a^	*p* value
Age groups		<0.001^∗^		<0.001^∗^
18–40 years	Referent		Referent	
41–60 years	4.98 (1.64–15.14)	0.005^∗^	4.69 (1.55–14.23)	0.006^∗^
61–80 years	10.42 (3.56–30.51)	<0.001^∗^	8.94 (3.04–26.33)	<0.001^∗^
81+ years	15.61 (5.10–47.80)	<0.001^∗^	12.72 (4.10–39.40)	<0.001^∗^
Male sex	1.55 (1.04–2.32)	0.031^∗^	1.70 (1.13–2.56)	0.012^∗^
Indigenous	3.23 (1.40–7.43)	0.006^∗^	3.12 (1.36–7.18)	0.007^∗^
Cancer history	0.52 (0.32–0.85)	0.009^∗^	0.52 (0.32–0.84)	0.008^∗^
PN	2.38 (1.56–3.61)	<0.001^∗^	2.26 (1.48–3.45)	<0.001^∗^
Surgeon past foot treatment	6.01 (2.74–13.18)	<0.001^∗^	5.16 (2.32–11.47)	<0.001^∗^

Model 1 results	Pseudo *R*^2^: 0.213 Omnibus: df = 8, *p* < 0.001	Missing: 11 (1.5%); H&L: *p* = 0.498	Pseudo *R*^2^: 0.222 Omnibus: df = 9, *p* < 0.001	Missing: 11 (1.5%); H&L: *p* = 0.038

^∗^ *p* < 0.05. ^a^Adjusted for identified confounder of past podiatry treatment; pseudo *R*^2^: Nagelkerke *R*^2^; omnibus: omnibus tests of model coefficients; df: degrees of freedom; missing: excluded cases with any missing data; H&L: Hosmer and Lemeshow test; PN: peripheral neuropathy.

**Table 7:** Independent factors associated with peripheral neuropathy (odds ratios [95% CI]).

Risk factor	Unadjusted	*p* value	Adjusted^a^	*p* value
Age groups		0.007^∗^		0.008^∗^
18–40 years	Referent		Referent	
41–60 years	2.93 (1.07–8.01)	0.037^∗^	2.77 (1.01–7.62)	0.048^∗^
61–80 years	4.66 (1.80–12.08)	0.002^∗^	4.55 (1.75–11.80)	0.002^∗^
81+ years	4.73 (1.70–13.15)	0.003^∗^	4.42 (1.59–12.32)	0.004^∗^
Diabetes	3.91 (2.57–5.97)	<0.001^∗^	3.94 (2.55–6.07)	<0.001^∗^
Mobility impairment	3.37 (2.22–5.11)	<0.001^∗^	3.41 (2.24–5.20)	<0.001^∗^
PAD	1.93 (1.25–2.99)	0.003^∗^	2.08 (1.33–3.25)	0.001^∗^
Outside footwear worn		0.017^∗^		0.055
Low risk	Referent		Referent	
Moderate risk	0.58 (0.29–1.15)	0.117	0.57 (0.28–1.13)	0.108
High risk	0.71 (0.45–1.11)	0.132	0.72 (0.45–1.14)	0.158
No footwear	3.99 (1.19–13.35)	0.025^∗^	3.01 (0.85–10.65)	0.088

Model 1 results	Pseudo *R*^2^: 0.284 Omnibus: df = 9, *p* < 0.001	Missing: 15 (2.0%); H&L: *p* = 0.100	Pseudo *R*^2^: 0.289 Omnibus: df = 13, *p* < 0.001	Missing: 33 (4.5%); H&L: *p* = 0.189

^∗^ *p* < 0.05. ^a^Adjusted for identified confounder of geographical remoteness; pseudo *R*^2^: Nagelkerke *R*^2^; omnibus: omnibus tests of model coefficients; df: degrees of freedom; missing: excluded cases with any missing data; H&L: Hosmer and Lemeshow test; PAD: peripheral arterial disease.

**Table 8:** Independent factors associated with foot deformity (odds ratios [95% CI]).

Risk factor	Unadjusted	*p* value	Adjusted^a^	*p* value
Age groups		<0.001^∗^	No confounders identified	
18–40 years	Referent			
41–60 years	1.76 (0.62–4.99)	0.289		
61–80 years	4.67 (1.79–12.17)	0.002^∗^		
81+ years	5.68 (2.05–15.71)	0.001^∗^		
Mobility impairment	2.04 (1.35–3.08)	0.001^∗^		
PN	2.20 (1.44–2.36)	<0.001^∗^		
Podiatry past foot treatment	2.06 (1.36–3.12)	0.001^∗^		

Model 1 results	Pseudo *R*^2^: 0.233 Omnibus: df = 6, *p* < 0.001	Missing: 32 (4.4%); H&L: *p* = 0.938		

^∗^ *p* < 0.05. ^a^No confounders were identified; pseudo *R*^2^: Nagelkerke *R*^2^; omnibus: omnibus tests of model coefficients; df: degrees of freedom; missing: excluded cases with any missing data; H&L: Hosmer and Lemeshow test; PN: peripheral neuropathy.
